# Draft Whole-Genome Sequences of *Escherichia fergusonii* Strains Isolated from Beef Trim (GTA-EF02), Ground Beef (GTA-EF03), and Chopped Kale (GTA-EF04)

**DOI:** 10.1128/genomeA.00185-16

**Published:** 2016-04-14

**Authors:** Paul Manninger, Adam Koziol, Catherine D. Carrillo

**Affiliations:** Canadian Food Inspection Agency, Government of Canada, Ottawa, Canada

## Abstract

*Escherichia fergusonii* is a Gram-negative, rod-shaped, non-spore-forming member of the *Enterobacteriaceae* family and is a bacterium with both biotechnological applications and implication in human clinical disease. Here, we report the draft genome sequences of three isolates of *E. fergusonii* from beef trim (GTA-EF02), ground beef (GTA-EF03), and chopped kale (GTA-EF04).

## GENOME ANNOUNCEMENT

*Escherichia fergusonii* is ubiquitous in nature, having been isolated from soil, water, plants, and animals. This organism was originally classified as enteric group 10, until it was recognized as a new bacterial species via DNA hybridization in 1985 (1). *E. fergusonii* is closely related to *Escherichia coli*, with which it exhibits a similar biochemical profile(s). It is also often misidentified as *Salmonella* due to a similar appearance on MacConkey agar (2, 3). Clinically, it is an opportunistic pathogen that was originally isolated from human blood samples, and it has since been implicated in human and animal disease (4, 5). Industrial applications include bioremediation (6) and hydrogen production (7).

*E. fergusonii* strains were recovered from food samples, including beef trim (GTA-EF02), ground beef (GTA-EF03), and chopped kale (GTA-EF04). Genomic DNA was isolated from overnight cultures grown on nutrient agar, using the Maxwell 16 cell DNA purification kit (Promega, Madison, WI). Sequencing libraries were constructed using the Nextera XT DNA sample preparation kit (Illumina, Inc., San Diego, CA), and paired-end sequencing was performed with the Illumina MiSeq platform, using 600-cycle MiSeq reagent kits (version 3). Sequencing errors in reads were corrected using Quake version 0.3, with a k-mer size of 15 (8), and assembled *de novo* using SPAdes version 3.5 (9). Contigs <1,000 bp were excluded from the analysis. Gene predictions and annotations were performed with the National Center for Biotechnology Information (NCBI) Prokaryotic Genome Annotation Pipeline (PGAP) (10). The assembly and annotation statistics are shown in Table 1.

**TABLE 1:** Genome assembly and annotation statistics for *E. fergusonii* isolates

Strain	Accession no.	Depth of coverage (×)	No. of contigs	Contig *N*_50_ (bp)	Assembly size (bp)	G+C content (%)	No. of CDSs^*a*^	No. of genes	No. of pseudogenes	No. of rRNAs	No. of tRNAs	Virulence gene(s)	Antimicrobial resistance
GTA-EF02	JZWQ00000000	114	22	403,657	4,764,067	49.85	4,470	4,691	116	11	75	*gad*, *iss*	Sulfonamide (*sul2*), aminoglycoside (*strA*, *strB*), tetracycline [*tet*(A)]
GTA-EF03	JZWN00000000	132	29	349,091	4,494,663	49.96	4,223	4,418	94	11	77	*gad*, *iss*, *prfB*	None predicted
GTA-EF04	JZWP00000000	164	39	336,937	4,644,753	49.86	4,443	4,641	92	13	77	*gad*	Aminoglycoside (*strA*, *strB*), tetracycline [*tet*(B)]

a CDSs, coding sequences.

Antimicrobial resistance (ResFinder [11]) and virulence profiles (VirulenceFinder [12]) were determined using tools freely available at http://cge.cbs.dtu.dk/services/, with the results shown in Table 1. Four resistance genes were found on the same 8.5-kb contig in strain GTA-EF02, which is predicted to be aminoglycoside, tetracycline, and sulfonamide resistant. The virulence genes identified included the *iss* (increased serum survival) gene in both beef isolates and the *prfB* (P-related fimbriae regulatory) gene in one of the beef isolates (13).

### Nucleotide sequence accession numbers.

This whole-genome shotgun project has been deposited at DDBJ/EMBL/GenBank under the accession numbers listed in Table 1. The versions described in this paper are the first versions.

## References

[1] Farmer JJ, Fanning GR, Davis BR, O’Hara CM, Riddle C, Hickman-Brenner FW, Asbury MA, Lowery VA, Brenner DJ 1985 *Escherichia fergusonii* and *Enterobacter taylorae*, two new species of *Enterobacteriaceae* isolated from clinical specimens. J Clin Microbiol 21:77–81. 3968204 10.1128/jcm.21.1.77-81.1985 PMC271579

[2] Funke G, Hany A, Altwegg M 1993 Isolation of *Escherichia fergusonii* from four different sites in a patient with pancreatic carcinoma and cholangiosepsis. J Clin Microbiol 31:2201–2203. 8370751 10.1128/jcm.31.8.2201-2203.1993 PMC265723

[3] Hariharan H, López A, Conboy G, Coles M, Muirhead T 2007 Isolation of *Escherichia fergusonii* from the feces and internal organs of a goat with diarrhea. Can Vet J 48:630–631. 17616063 PMC1876196

[4] Freney J, Gavini F, Ploton C, Leclerc H, Fleurette J 1987 Isolation of *Escherichia fergusonii* from a patient with septicemia in France. Eur J Clin Microbiol 6:78. doi:10.1007/BF02097203. 3569257

[5] Gaastra W, Kusters JG, van Duijkeren E, Lipman LJA 2014 Escherichia fergusonii. Vet Microbiol 172:7–12. doi:10.1016/j.vetmic.2014.04.016. 24861842

[6] Sriram MI, Gayathiri S, Gnanaselvi U, Jenifer PS, Mohan Raj S, Gurunathan S 2011 Novel lipopeptide biosurfactant produced by hydrocarbon degrading and heavy metal tolerant bacterium *Escherichia fergusonii* KLU01 as a potential tool for bioremediation. Bioresour Technol 102:9291–9295. doi:10.1016/j.biortech.2011.06.094. 21802283

[7] Farghaly A, Tawfik A, Danial A 2016 Inoculation of paperboard mill sludge versus mixed culture bacteria for hydrogen production from paperboard mill wastewater. Environ Sci Pollut Res Int 23:3834–3846. doi:10.1007/s11356-015-5652-7. 26498965

[8] Kelley DR, Schatz MC, Salzberg SL 2010 Quake: quality-aware detection and correction of sequencing errors. Genome Biol 11:R116. doi:10.1186/gb-2010-11-11-r116. 21114842 PMC3156955

[9] Bankevich A, Nurk S, Antipov D, Gurevich AA, Dvorkin M, Kulikov AS, Lesin VM, Nikolenko SI, Pham S, Prjibelski AD, Pyshkin AV, Sirotkin AV, Vyahhi N, Tesler G, Alekseyev MA, Pevzner PA 2012 SPAdes: a new genome assembly algorithm and its applications to single-cell sequencing. J Comput Biol 19:455–477. doi:10.1089/cmb.2012.0021. 22506599 PMC3342519

[10] Tatusova T, DiCuccio M, Badretdin A, Chetvernin V, Ciufo S, Li W 2013 Prokaryotic genome annotation pipeline. The NCBI handbook, 2nd ed. National Center for Biotechnology Information, Bethesda, MD.

[11] Zankari E, Hasman H, Cosentino S, Vestergaard M, Rasmussen S, Lund O, Aarestrup FM, Larsen MV 2012 Identification of acquired antimicrobial resistance genes. J Antimicrob Chemother 67:2640–2644. doi:10.1093/jac/dks261. 22782487 PMC3468078

[12] Joensen KG, Scheutz F, Lund O, Hasman H, Kaas RS, Nielsen EM, Aarestrup FM 2014 Real-time whole-genome sequencing for routine typing, surveillance, and outbreak detection of verotoxigenic *Escherichia coli*. J Clin Microbiol 52:1501–1510. doi:10.1128/JCM.03617-13. 24574290 PMC3993690

[13] Wragg P, La Ragione RM, Best A, Reichel R, Anjum MF, Mafura M, Woodward MJ 2009 Characterisation of *Escherichia fergusonii* isolates from farm animals using an *Escherichia coli* virulence gene array and tissue culture adherence assays. Res Vet Sci 86:27–35. doi:10.1016/j.rvsc.2008.05.014. 18585745

